# Self-Efficacy and Intention to Quit Vaping: A Systematic Review of Empirical Evidence Across Behavioural Studies

**DOI:** 10.21315/mjms-12-2025-898

**Published:** 2026-06-30

**Authors:** Nuranida Hani Ruslan, Che Julia Jailan, Nawwal Alwani Mohd Radzi, Amirah Azzeri, Norashikin Yusof

**Affiliations:** 1 Centre of Population Oral Health and Clinical Prevention Studies, Faculty of Dentistry, Universiti Teknologi MARA, Sungai Buloh Campus, Selangor Branch, Sungai Buloh, Selangor, Malaysia; 2 Ministry of Health, Putrajaya, Malaysia; 3 Public Health Unit, Faculty of Medicine and Health Sciences, Universiti Sains Islam Malaysia, Nilai, Negeri Sembilan, Malaysia

**Keywords:** self-efficacy, electronic nicotine delivery systems, smoking cessation

## Abstract

The increasing global prevalence of e-cigarette use, particularly among young adults, highlights the critical need for effective cessation strategies. This systematic review aims to map the conceptualisation, measurement and nature of the association between self-efficacy (SE) and intention to quit (ITQ) vaping across empirical studies. Searches were conducted across primary bibliographic databases (PubMed/MEDLINE, Scopus, Web of Science and Cochrane Library) until 1 November 2025. Additionally, other methods were employed, consisting of a grey literature search via Springer Link (finalised 1 November 2025) and targeted citation tracking through Google Scholar (finalised 12 November 2025). Review articles, qualitative-only studies, editorials and conference abstracts were excluded. Data were synthesised narratively, reporting regression coefficients (*β*) and odds ratios. Following PRISMA 2020 guidelines, 11 studies were included, all of which were cross-sectional (*n* = 11) and conducted mainly in the US (*n* = 8). Quality assessment using the Joanna Briggs Institute Checklist rated 10 studies as having a low risk of bias. SE was consistently conceptualised as confidence in quitting or resisting e-cigarette use, measured via single- and multi-item scales (α: 0.81, 0.98). ITQ reflected readiness or determination. Preliminary evidence suggests a predominantly positive association between these constructs (*β*: 0.16, 0.56), although the observed effects varied across studies. The evidence base exhibited substantial heterogeneity in study design, measurement approaches and study populations. Limitations include the predominance of cross-sectional designs and Western-based samples. Interventions should integrate SE enhancement strategies and future research should employ longitudinal, cross-cultural designs. This review received no financial or non-financial support. The protocol was registered with PROSPERO (CRD420251207425).

## Introduction

The global prevalence of e-cigarettes and vaping has increased dramatically over the past decade, particularly among young adults, raising public health concerns regarding nicotine dependence and potential long-term health consequences (1, 2). Current estimates indicate that there are approximately 82 million e-cigarette users worldwide (3). Within this population, the magnitude of intention to quit (ITQ) is substantial, with research showing that roughly 55.3% of young adult vapers express a clear ITQ within the next six months and 43.6% have made at least one quit attempt in the past year (4, 5). However, even with these high rates of intention and prior attempts, the rate of successful cessation remains disproportionately low compared to traditional tobacco. Despite the growing uptake of e-cigarettes, cessation efforts among e-cigarette users remain underexplored, especially in relation to psychological constructs such as self-efficacy (SE) and ITQ. SE, defined as an individual’s confidence in their ability to perform a specific behaviour (6), has been widely recognised as a key determinant of behaviour change in smoking and vaping cessation. Higher SE has been consistently linked to greater readiness to initiate quit attempts and persist through withdrawal or environmental challenges (7, 8). Behavioural theories, such as the Social Cognitive Theory (SCT), emphasise the interplay of personal, behavioural and environmental factors in shaping SE and subsequent health behaviours, providing a framework for understanding the mechanisms through which confidence influences quitting behaviour (6, 9).

Intention to quit vaping reflects an individual’s commitment or plan to cease e-cigarette use within a defined timeframe, often operationalised as readiness to quit within the next 30 days, six months or year (10). Unlike motivation, which captures general desire or willingness to change behaviour without specifying a timeline, ITQ involves concrete planning and anticipatory commitment, bridging the gap between desire and action (11). Examining SE and ITQ together is essential because unless accompanied by clear intentions and planning, confidence alone may not translate into actual cessation. Behavioural theories, such as SCT, the Theory of Planned Behaviour (TPB) and the Transtheoretical Model, suggest that higher perceived capability enhances the formation of concrete quit intentions, which in turn increases the likelihood of initiating cessation attempts and maintaining abstinence (12, 13). Empirical studies support this connection: individuals with higher SE are more likely to form actionable ITQ, whereas low SE can limit the translation of motivation into concrete quit plans, particularly among dual users or those exposed to social or environmental cues that reinforce vaping behaviour (3, 14–16).

Although the role of SE in traditional smoking cessation has been well documented in existing systematic reviews, these findings cannot be directly extrapolated to e-cigarette use. Vaping presents unique behavioural challenges, including nicotine delivery variations, distinct social usage patterns and a lack of standardised clinical cessation guidelines, distinguishing it from combustible tobacco. Existing reviews in the vaping field have primarily focused on the health risks of e-cigarettes or their efficacy as a tool for quitting smoking, rather than the psychological mechanisms required to quit vaping. Consequently, evidence on how SE and ITQ are operationalised and examined within the context of e-cigarette cessation remains limited. Despite its theoretical and practical importance, empirical research examining SE and ITQ concurrently among e-cigarette users remains limited and heterogeneous. Existing studies have primarily relied on cross-sectional surveys, with variable measurement approaches and inconsistent operationalisation of constructs, often focusing on young adults in Western populations (5, 17, 18). Single- and multi-item instruments have been used to assess both SE and ITQ, with studies guided by behavioural models reporting positive associations between confidence to quit and intention to cease vaping. However, few studies have explored the differences between exclusive e-cigarette users, dual users and former smokers, and limited longitudinal data exist to examine how SE and ITQ evolve over time.

Distinguishing vaping cessation from traditional smoking cessation is critical because e-cigarette users often face unique physiological and social drivers. For instance, the high-frequency puffing patterns and varying nicotine concentrations in electronic nicotine delivery systems (ENDS) create different dependence profiles than combustible cigarettes. Furthermore, the social vaping culture and the perception of e-cigarettes as harm-reduction alternatives can influence an individual’s SE and ITQ in ways not observed in traditional tobacco users. Therefore, this review treats vaping as a distinct behavioural entity rather than a subset of tobacco use. Understanding these constructs has significant implications for research, policy and practice. Behavioural theories highlight a strong connection between SE or confidence in one’s ability to quit and ITQ, reflecting readiness to change. However, evidence in the context of vaping cessation remains fragmented, with limited studies explicitly examining this relationship rather than measuring each construct separately. The rationale for this systematic review is to clarify conceptual ambiguity and methodological inconsistency. Clarifying the conceptualisation and measurement of SE and ITQ facilitates reliable assessment, cross-study comparisons and the design of longitudinal or intervention studies targeting vaping cessation. Evidence on SE and ITQ can guide the development of targeted cessation programmes and public health campaigns that enhance perceived control and support quitting behaviour from a policy perspective. In clinical and public health practice, integrating SE assessment allows practitioners to tailor motivational strategies and reinforce quit planning, ultimately improving cessation outcomes.

The primary objective of this study is to map the nature and extent of the association between SE and intention to quit vaping. This review provides a comprehensive overview of how self-belief in one’s ability to quit relates to the intention to cease e-cigarette use by synthesising both quantitative correlations and descriptive evidence. Secondary objectives include summarising the measurement instruments used and identifying key research gaps in the existing empirical literature, particularly regarding the frequency of formal statistical testing of the SE–ITQ relationship. Grounding interventions in these findings ensures that programmes address both psychological determinants and actionable planning, bridging the gap between intention and sustained abstinence. Taken together, a comprehensive understanding of SE and ITQ is essential for advancing both the science and practice of e-cigarette cessation, enabling the design of interventions that strengthen confidence while simultaneously fostering concrete intentions to quit vaping.

## Methods

### Protocol Registration

The protocol for this systematic review was developed a priori and registered with the International Prospective Register of Systematic Reviews (PROSPERO) under the registration ID: CRD420251207425. The protocol can be seen through the link https://www.crd.york.ac.uk/PROSPERO/view/CRD420251207425. The review was conducted in accordance with the Preferred Reporting Items for Systematic Reviews and Meta-Analyses (PRISMA 2020) guidelines.

### Information Sources and Search Strategy

Comprehensive electronic searches were conducted across primary bibliographic databases, including PubMed/MEDLINE, Scopus, Web of Science and the Cochrane Library, for articles from inception until 1 November 2025. Concurrently, a manual search for grey literature and academic reports was conducted via the Springer Link platform (finalised 1 November 2025). A final manual search was conducted on 12 November 2025 using Google Scholar to ensure a complete evidence base. Backward citation tracking was performed by screening the reference lists of all included studies and forward citation tracking was conducted by examining articles that cited the included studies.

This systematic review was guided by the following primary research question: How have empirical studies conceptualised and measured SE and intention to quit vaping, and what is the nature of the association between these two constructs? To further expand the view, the following sub-questions were formulated:

- What instruments, reliability measures and Likert-type scales are used to assess SE and intention to quit vaping?
- To what extent do existing studies report an association between SE and intention to quit vaping (either through statistical testing or descriptive findings)?
- What types of evidence (e.g. quantitative correlations or descriptive observations) are available to understand this relationship?
- What is the direction and nature of the relationship between SE and intention to quit vaping as reported across studies?

In addition to addressing the above-mentioned questions, this review aims to provide evidence that can inform future research directions, policy formulation and clinical or public health practice.

The search strategy combined controlled vocabulary terms, including Medical Subject Headings (MeSH) where available, with free-text keywords to capture variations in terminology for both SE and vaping-related outcomes. For example, terms such as “SE,” “confidence to quit,” and “perceived control” were combined with e-cigarette-related keywords such as “vaping,” “e-cigarette,” “electronic cigarette,” “ENDS,” “JUUL,” and “pod devices.” To capture intention-related constructs, additional keywords such as “ITQ,” “readiness to quit,” “motivation to quit,” and “cessation” were included. Boolean operators (AND/OR) and truncation symbols were applied to ensure comprehensive retrieval across multiple databases, while adaptations were made to accommodate each database’s syntax. Appendix 1 provides the detailed search strategy, including the combination of keywords, MeSH terms and Boolean operators used across all databases.

### Study Selection and Inclusion Criteria

This review included quantitative studies employing cross-sectional, longitudinal or interventional designs that examined the relationship between SE and intention to quit vaping or e-cigarette use. Eligible studies included at least one measure of SE related to quitting or reducing vaping and a measure reflecting intention, motivation or readiness to quit. The constructs were assessed within the same study, although examining their association was not a prerequisite for inclusion. Validated multi-item instruments and single-item measures were accepted. No restrictions were imposed on the publication year, language or geographical location to ensure comprehensive coverage of all relevant evidence. Studies that involved current, former or dual users of e-cigarettes and provided quantitative data examining associations, correlations or predictive relationships between SE and ITQ were included.

Review articles, qualitative-only studies, editorials and conference abstracts were excluded because they did not provide extractable quantitative data. Two reviewers (NHR and CJJ) conducted all search activities collaboratively. The search results were exported into Microsoft Excel 2021 for reference management, duplicate removal and screening without the use of automation tool. After eliminating duplicates and clearly irrelevant records, both reviewers independently reviewed the titles and abstracts based on the predefined eligibility criteria. Full-text articles of potentially relevant studies were then retrieved and assessed independently by the same reviewers for final inclusion. Any discrepancies during the search, screening or eligibility assessment were resolved through discussion between NHR and CJJ, with consultation with a third reviewer (NY) when necessary.

### Data Extraction and Quality Assessment

A customised data extraction form was developed in Microsoft Excel 2021 to systematically capture the included studies’ methodological and conceptual characteristics. The extracted variables included general study characteristics (author, publication year, country and study design), participant characteristics (sample size, mean or range of age, gender distribution and user type such as exclusive vapers, dual users or ex-vapers) and theoretical frameworks underpinning the study (e.g. SCT, TPB, Health Belief Model [HBM], Transtheoretical Model). Measurement details were also recorded, including the conceptualisation of SE and ITQ, instruments used, scale or response format, reliability indices (e.g. Cronbach’s alpha), mean scores or percentages and reported statistical significance. The relationship between SE and ITQ, defined as any statistical or theoretical association reported between these constructs, was the primary outcome for which data were sought. All compatible results related to this outcome were extracted, including findings from correlation, regression, path analysis and structural equation modelling, regardless of time point or measurement scale. To ensure the integrity of the evidence, no assumptions regarding missing or ambiguous data were made. If specific indices or details were not explicitly stated, they were recorded as “not reported.” Study quality was assessed using appraisal tools appropriate to the included studies’ design. As all studies included in the final review were analytical cross-sectional studies, the Joanna Briggs Institute (JBI) Checklist for Analytical Cross-Sectional Studies was used to assess methodological quality (19).

Data extraction and quality assessment were conducted independently by NHR and CJJ, with discrepancies resolved through discussion or consultation with a third reviewer (NY). Potential reporting biases were addressed by reviewing each study for the completeness of outcomes related to SE and intention to quit smoking. Studies that lacked key statistical estimates were recorded and no attempts to impute missing data were made. The possibility of selective outcome reporting was considered when assessing the quality of the study and interpreting the findings. Any discrepancies between the reviewers were resolved iteratively through discussion until a consensus was reached. Baseline inter-rater agreement statistics (e.g. pre-consensus Cohen’s Kappa) were not calculated because disagreements were addressed and resolved during the iterative data extraction process.

### Data Synthesis and Effect Measures

Data extracted from the included studies were synthesised narratively to provide an integrated overview of how SE and intention to quit vaping were conceptualised, measured and analysed across empirical research. The additional variables extracted included study characteristics, participant demographics, theoretical frameworks, measurement instruments and reliability indices.

Effect measures extracted for the primary outcome, the relationship between SE and ITQ, included regression coefficients (*β*), unstandardised B values, odds ratios (ORs) and correlation coefficients (*r*), depending on the statistical approach reported in each study. *P*-values were recorded to determine the statistical significance of these relationships. All relevant estimates were extracted in studies reporting multiple models or waves, with priority given to the most comprehensive or adjusted values. Logistic regression analyses provided ORs indicating the change in the odds of intending to quit per unit increase in SE, whereas hierarchical regression analyses reported *β* coefficients reflecting the strength and direction of the association. Descriptive statistics, such as mean SE and ITQ scores or percentages, were also recorded when available.

To determine eligibility for synthesis, studies were assessed against predefined inclusion criteria, including population, outcomes and study design. Study characteristics, measurement instruments and analytical approaches were tabulated to identify suitable studies for comparison. Data were standardised, including harmonising scale directions and converting reported percentages into proportions. Missing or unclear statistical information was noted as “not reported.”

Results were presented in summary tables capturing participant and methodological characteristics, measurement and reliability details of SE and ITQ instruments and the statistical relationship between SE and ITQ, including direction, strength and significance. A narrative synthesis was conducted due to heterogeneity in study designs, populations, measurement scales and analytical approaches. The synthesis focused on patterns in the conceptualisation and measurement of SE and ITQ, frequency and method of testing SE–ITQ associations and strength and direction of reported relationships. A meta-analysis was not performed due to insufficient homogeneity in the study measures. Although quantitative pooling via meta-analysis was considered, a narrative synthesis was considered more appropriate. This decision was based on the methodological heterogeneity of the included studies, specifically the use of incompatible outcome metrics, such as binary ORs (16) versus continuous regression coefficients (3) and the use of adjusted values from disparate multivariate models, which limit the validity of standardised transformations (20). Where possible, results were described separately for exclusive vapers, dual users and ex-vapers. Sensitivity of findings was considered by comparing higher versus lower methodological quality studies and by noting the consistency of associations across different analytical models and time points.

## Results

### Study Selection

The study selection process is detailed in the PRISMA 2020 flow diagram (Figure 1). A total of 1,143 records were identified through primary bibliographic databases (PubMed/MEDLINE, Scopus, Web of Science and the Cochrane Library), with the final search completed on 1 November 2025. Following the removal of 110 duplicates, a total of 1,033 records were screened by title and abstract. Concurrently, studies were identified via other methods, including a search for grey literature in the Springer Link platform (*n* = 355) and a supplementary search of Google Scholar (*n* = 10), yielding 365 additional records. No duplicates (*n* = 0) were removed from these 365 records, as they were pre-cleaned during the identification phase. However, all 365 were subsequently excluded during the title and abstract screening stage because they failed to meet the eligibility criteria. From the database search, 27 reports were sought for retrieval and 100% were successfully retrieved for full-text assessment. Following the eligibility review of these 27 reports, 16 were excluded for the following reasons: irrelevant constructs (*n* = 8), focus unrelated to vaping cessation (*n* = 7) and ineligible study type (*n* = 1). Ultimately, 11 studies met all inclusion criteria and were included in the final systematic review.

### Study Characteristics

Table 1 summarises the study characteristics of the 11 included studies. All 11 included studies employed a cross-sectional design. While two of these studies collected data at multiple time points (waves) (5, 21), they classified them as repeated cross-sectional surveys because independent samples were used at each wave rather than tracking the same participants. Geographically, the research was predominantly conducted in the United States (*n* = 8), with the remaining studies conducted in the Netherlands (*n* = 2) and Australia (*n* = 1). Sample sizes varied significantly, ranging from 40 to 1,061 participants. The target populations primarily consisted of young adults (mean age ~20 to 30 years), although a few studies included older adults. Participants across the evidence base represented a diverse range of users, including exclusive e-cigarette users, dual users of cigarettes and e-cigarettes and former smokers.

Theoretical frameworks were consistently applied to examine the psychological drivers of cessation. The SCT was the most frequently used framework to assess personal confidence and perceived control. Other identified models included the Multi Theory Model (MTM), the TPB and the I-Change Model (18, 22). Collectively, these studies aimed to identify key cessation predictors to inform targeted public health communication strategies.

### Quality Appraisal

The JBI Checklist for Analytical Cross-Sectional Studies (23) for cross-sectional studies (*n* = 11), which comprises eight appraisal items, was used to evaluate the methodological quality of the included studies. Each item was scored as 1 for “Yes” (criterion met) and 0 for “No” or “Unclear” (criterion not met or unclear). Consistent with methods applied in previous research (24), studies were considered to have a low risk of bias if their total score was 70%, a moderate risk of bias if the score ranged from 50% to 69% and a high risk of bias if the score was 50%.

Table 2 illustrates the details of each study’s quality appraisal outcomes. Most studies demonstrated a low risk of bias, consistent adherence to inclusion criteria, appropriate subject selection, identification and control of confounding factors and robust statistical analysis. Overall, 10 studies were rated as low risk, while one study was rated as moderate risk (14), mainly due to missing data on confounding strategies and incomplete outcome measures or exposure data. Overall, most studies demonstrated high methodological quality, with only minor limitations observed.

### Conceptualisation of SE

Across the included studies (Table 3), SE was consistently conceptualised as participants’ perceived confidence in their ability to quit or resist e-cigarette use. This construct was operationalised in various ways, such as “quit confidence” (5), “confidence to resist vaping in challenging situations” (17, 22, 23) or “confidence in the ability to abstain from vaping and smoking” (15). Other studies used SE as a subconstruct within broader theoretical models, for example, the behavioural confidence component of the MTM (18). Some authors assessed the perceived impact of intervention messages related to confidence (4), while others focused on momentary confidence to quit (3, 16). Meanwhile, Jun et al. (21) explored participants’ agreement with statements reflecting confidence to stop using e-cigarettes, linking these beliefs with cessation planning and attempts. Overall, SE in these studies broadly referred to the cognitive appraisal of one’s ability to refrain from or discontinue e-cigarette use in specific contexts, whether emotional, social or situational.

### Measurement Approaches and Reliability of SE

SE assessment commonly employed Likert-type scales ranging from 5-point to 10-point formats, with higher scores indicating stronger confidence to quit or resist vaping. Multi-item measures were prevalent, such as the 16-item 5-point scale assessing confidence in resisting vaping across multiple contexts (α = 0.95) (22) and the 19-item modified SE scale (17, 23). Studies applying theoretical frameworks reported excellent internal consistency for the MTM behavioural confidence subscale (α = 0.91) (18). Single-item scales, including 10-point Likert or categorical ratings (3, 5, 16), were also used to provide concise measure assessments of quit confidence. Reliability values ranged from α = 0.81 to 0.98, reflecting high internal consistency among the instruments. The mean SE scores varied across the samples, typically ranging from 2.05 to 7.59. Exclusive e-cigarette users generally reported higher SE compared with dual users (14, 15), while exposure to social networking site marketing content was linked to markedly lower SE (17). Although measurement approaches differed, most instruments demonstrated robust reliability and consistent use of Likert-type response formats to quantify confidence in cessation behaviour.

### Conceptualisation of ITQ

The conceptualisation of intention to quit vaping varied considerably across the included studies, as illustrated in Table 4. Several studies have used the ITQ as an indicator of readiness to quit within specific timeframes, reflecting participants’ planned cessation behaviour. Hobkirk et al. (5) used a single-item question to ask JUUL users whether they planned to quit within a year, continued using or were uncertain, with 22.6% reporting an ITQ. Similarly, two studies (4, 14) conceptualised readiness to quit as an ITQ, employing categorical scales ranging from “within the next 30 days” to “not thinking about quitting.” Both studies reported low levels of readiness: one study (4) found that only 16.3% of participants planned to quit within 30 days, while another study (14) noted that over 44% of both exclusive e-cigarette users and dual users were not contemplating cessation.

Romijnders et al. (15) employed two ITQ constructs: agreement with quitting statements within six months and semantic-differential items, with dual users consistently showing higher mean scores than exclusive vapers. Another two studies (17, 23) also defined intention as a motivational construct, including items such as “During the next six months, I intend to quit using e-cigarettes.” Both studies demonstrated high internal consistency (α = 0.98) and found that social and environmental factors, such as exposure to online e-cigarette content and peer influence, influenced the ITQ.

### Measurement Approaches and Scale Formats for ITQ

The studies applied various measurement formats, including categorical, binary and Likert-type scales, to assess ITQ. Hobkirk et al. (5) a categorical three-point scale, whereas some studies (4, 14) employed four-point categorical formats. These categorical approaches focused on timing-based readiness, aligning with the Transtheoretical Model. In contrast, Likert-type or continuous scales were used to capture the intensity of quitting intention, as demonstrated in two studies (17, 22).

We also used binary and time-based measures. Wang-Schweig et al. (16) measured the ITQ as a binary (yes/no) response to whether participants were seriously considering quitting within six months. They found that 37.3% of smokers and dual users reported such intentions, compared with only 15.4% of exclusive vapers. Jun et al. (21) utilised a five-category measure of cessation intent and showed slight increases in quit plans over time, from 20.0% to 25.1% among those planning to quit within a month and from 24.2% to 30.8% among those planning to quit within six months, although these changes were not statistically significant.

### Psychometric Evaluation and Measurement Implications of the SE and ITQ

Although the internal consistency for the multi-item scales used in the included studies was high (α: 0.81, 0.98), several psychometric limitations warrant discussion. First, many instruments were adapted directly from smoking cessation literature without undergoing formal validation for the unique vaping behavioural context. For instance, Rahman et al. (3) utilised the 11-point Contemplation Ladder, a tool originally validated for combustible cigarette cessation, while Sharma et al. (18) employed the MTM “surety scale.” The transition of these tools from tobacco to e-cigarettes assumes that the psychological drivers of quitting are identical, potentially overlooking vaping-specific factors such as high-frequency “grazing” or varying nicotine delivery systems.

Second, there was significant heterogeneity in scale formats, which introduces measurement error risks. For example, Hobkirk et al. (5) used a 10-point Likert scale for quit confidence, whereas Sharma et al. (18) used a 5-point Likert scale. Similarly, the ITQ was measured using formats ranging from a 3-point categorical scale (5) to a binary “Yes/No” format in other studies. Such discrepancies limit the ability to perform standardised comparisons and may lead to an overestimation or underestimation of the statistical association between SE and ITQ. Finally, because all data across the 11 studies were self-reported, an inherent risk of social desirability bias exists, which may artificially inflate reported confidence or intention levels.

### Frequency of Association Testing

Of the 11 studies that measured both SE and intention to quit vaping, only 4 (36.4%) explicitly tested a statistical association between these two constructs (3, 16, 18, 21). Table 5 presents the details of these tests. This association testing was often conducted within established behavioural frameworks: Rahman et al. (3) tested the link under the SCT, Sharma et al. (18) used the MTM and Jun et al. (21) applied the HBM. The remaining seven studies measured both constructs but either used ITQ as a standalone outcome or analysed SE as an independent predictor of other cessation behaviours, thus failing to explicitly test the theoretically hypothesised pathway between them.

### Analytical Approaches Used to Examine Relationships

The four studies that examined the relationship between SE (or the related construct, behavioural confidence) and ITQ primarily used regression analysis to evaluate predictive power (Table 5). Three studies (3, 18, 21) employed hierarchical regression analysis, a robust method used to evaluate the additional explanatory power of SE, often entered in the final block after controlling for various covariates, including sociodemographic, e-cigarette dependence and other behavioural model variables. For instance, Rahman et al. (3) used this approach to assess the incremental contribution of SCT constructs, including SE, on quit intention. Wang-Schweig et al. (16) used multiple logistic regression analysis to determine the change in the odds of intending to quit based on unit increases in SE scores. The application of these advanced regression models signifies a focus on determining predictive causality within theoretical frameworks, such as SCT, the HBM and the MTM.

### Direction and Nature of SE–ITQ Relationship

Of the four studies that tested the SE–ITQ relationship, three reported significant positive associations. Sharma et al. (2022) (18) found that behavioural confidence was the strongest predictor for quit initiation (*β* = 0.560; *P* < 0.001) in Model 3, while Rahman et al. (3) reported smaller but significant independent associations (*β* = 0.164; *P* < 0.001). However, Jun et al. (21) observed a negative association (*β* = –0.153; *P* < 0.05) in their Wave 2 sample. Although moderation was not clearly tested, the authors attributed this to an optimistic bias, where high confidence in one’s ability to quit easily may paradoxically hinder the formation of concrete quit plans.

From a clinical perspective, these effect sizes suggest that although SE is a relevant factor, its impact is small to moderate. The wide range of coefficients (*β* = 0.16, 0.56) indicates that SE likely functions as one part of a complex behavioural process. Given that only four studies (k = 4) clearly tested this link, the evidence should be regarded as preliminary rather than definitive proof of SE as a central predictor.

## Discussions

### Primary Findings

The included studies demonstrated wide heterogeneity in study design, sample demographics and analytical approaches, reflecting the evolving and multifaceted nature of research on SE and ITQ e-cigarette or vaping use. However, a major limitation of the current evidence base is the exclusive reliance on cross-sectional designs across all 11 included studies. While a relationship between SE and ITQ was consistently observed, the nature of these data precludes any causal inference. It is not yet clear whether higher SE precedes the formation of an ITQ or whether the process of deciding to quit is associated with an increase in a user’s confidence. The participants were predominantly young adults (mean age, 19 to 42 years), which is consistent with previous studies indicating that vaping is largely concentrated among youth and young adults (1, 25, 26).

Although some studies reported a predominance of male among dual users or smokers, gender distribution was generally balanced, aligning with broader trends in tobacco product use. Qualitative and mixed-methods studies (14) provided additional contextual insight into motivational patterns, complementing the quantitative measures of confidence and readiness to quit. Self-reported measures were a common limitation across studies, introducing potential recall or social desirability bias; however, internal reliability was enhanced by the use of validated scales (e.g. MTM, I-Change and 19-item SE scales).

SE was consistently conceptualised as confidence in the ability to resist or quit using e-cigarettes. Scores varied widely, with exclusive vapers often reporting higher confidence than dual users (14, 15), though some studies observed lower confidence among exclusive vapers compared with smokers or dual users (16). Social and environmental factors, including exposure to e-cigarette marketing and participation in vaping-related social media communities, were associated with lower SE, underscoring the influence of peer and environmental contexts (17, 23).

Dual users generally exhibited higher ITQ than exclusive vapers (15, 22), a finding that may be linked to the perceived cumulative health risks associated with maintaining both cigarette and e-cigarette use. Dual users are often more aware of the compounded harm from concurrent exposure to traditional tobacco smoke and vaporised nicotine products, which can increase their motivation to quit one or both products. This pattern suggests that quit intentions may stem not only from health awareness but also from ambivalence or dissatisfaction with maintaining dual use. Moreover, the presence of traditional smoking behaviour within this group may make them more likely to engage with cessation services or consider complete abstinence compared with exclusive e-cigarette users, who may perceive vaping as a safer or more acceptable alternative.

Jun et al. (21) reported a statistically significant inverse association (*β* = −0.153) in their Wave 2 sample. This may be theoretically interpreted as an “optimistic bias,” where high confidence in the ability to quit easily may paradoxically be linked to a reduced urgency to form concrete quit plans. This highlights the complex nature of SE in vaping, where confidence alone may not be sufficient to increase intention without a high level of perceived importance or readiness.

SE is a well-established construct in smoking cessation, but its application to vaping requires a distinct perspective. Vaping is used differently from traditional smoking, with patterns such as frequent use and varying nicotine levels, which may influence how and why users decide to quit. Therefore, findings from traditional smoking cessation meta-analyses or other smoking cessation studies cannot be directly extrapolated to vaping cessation contexts. Our results suggest that SE generally correlates with quit intentions, but the evidence remains preliminary due to the limited number of association studies (k = 4) and the potential for optimistic bias to negatively affect immediate quit planning.

### Strengths and Limitations

The primary strength of this review is its focused examination of SE across diverse populations using validated instruments and advanced statistical analyses, such as hierarchical regression, to control for confounding variables. This methodological rigour provides a clear overview of the relationship between confidence and quit intentions in the current vaping literature.

However, several critical limitations must be acknowledged. First, all included studies (*n* = 11) utilised cross-sectional designs, including repeated cross-sectional surveys with independent samples, which precludes any determination of causality or temporal precedence between SE and ITQ. Second, only four studies explicitly conducted statistical association testing (k = 4), indicating that the inferential evidence base remains relatively small.

Third, while a meta-analysis with k = 4 is technically feasible, a narrative synthesis was maintained due to significant methodological and clinical heterogeneity. The available effect measures comprised adjusted coefficients (ORs and *β* values) from multivariate models rather than raw data. Pooling these is statistically hazardous as they represent leftover variance after controlling for disparate sets of covariates. Furthermore, the operationalisation of ITQ varied from binary choices to continuous scales, representing distinct psychological constructs, making a choice versus intensity of motivation, which are not clinically comparable. Given the vast contextual differences between clinical recovery populations, including youth samples during the COVID-19 pandemic, a pooled estimate would likely yield misleadingly high heterogeneity (I-squared) and lack a clear clinical interpretation.

Fourth, significant measurement heterogeneity was observed across the 11 studies, with tools ranging from binary Yes/ No formats to 10-point scales, many of which were adapted from smoking literature without specific validation for vaping. Fifth, the reliance on self-reported measures introduces potential social desirability and recall biases. Sixth, the evidence is predominantly limited to Western populations, particularly the United States (*n* = 8), thus limiting generalizability to non-Western cultural or regulatory contexts. Finally, as with any systematic review, there is an inherent risk of publication bias, where studies with non-significant or negative findings may be underrepresented in the published literature.

### Implications for Research, Policy and Practice

These findings suggest a significant association between SE and ITQ among individuals who vape. Therefore, interventions designed to strengthen SE are potential components for enhancing the effectiveness of vaping cessation programmes. While literature in the broader tobacco control field suggests a link between confidence and abstinence, further vaping-specific longitudinal research is required to confirm these patterns in the context of e-cigarette use. Thus, enhancing confidence in one’s ability to quit may serve as a relevant correlate of successful cessation intentions.

Incorporating SE enhancement techniques, such as motivational interviewing, cognitive-behavioural skills training, self-monitoring and goal setting, into cessation programmes can increase the perceived control over quitting behaviour. Motivational interviewing promotes autonomy and intrinsic motivation, which can strengthen SE (27). Emerging digital health interventions, such as mobile health (mHealth) applications, chatbots and gamified behaviour change platforms, offer promising avenues for sustaining SE over time (28, 29). For example, gamified features such as reward systems, progress badges or virtual support groups, may enhance user motivation and perceived competence, particularly among younger adults who represent the predominant vaping demographic.

From a policy perspective, it is equally critical to strengthen environmental and structural supports. Exposure to pro-vaping marketing and social media content has been associated with lower SE and reduced quitting motivation (15, 17). Thus, policies that regulate digital marketing, restrict youth-targeted advertisements and promote evidence-based cessation resources could indirectly enhance the SE of the population to quit. Furthermore, embedding vaping cessation programmes into primary care, dental clinics and university health services would ensure broader accessibility and support continuity. Despite preliminary evidence linking SE and ITQ, gaps remain regarding how these constructs evolve across diverse populations. Thus, future research should adopt longitudinal and mixed-method designs to portray the dynamic interplay among SE, intention and actual behaviour change.

Moreover, research across different sociocultural settings is required to examine how cultural background, family dynamics and community perceptions of vaping may influence the functioning of SE within behavioural models. Research should also explore intersectional factors, such as gender, socioeconomic status and psychological comorbidities, which may be associated with SE and ITQ. Integrating psychometric advancements, such as item response theory and cross-cultural validation of SE and ITQ measures, would further strengthen future studies’ methodological rigour.

Clinicians and public health practitioners should emphasise SE assessment and reinforcement as a core component of cessation counselling. Incorporating structured SE assessments (e.g. SE for Smoking Cessation Scale or MTM Behavioural Confidence Subscale) can help practitioners tailor feedback and interventions according to patients’ confidence levels. Additionally, collaborative goal setting, progressive skill building and social support mobilisation can empower patients to translate intention into sustained behaviour change. Dental professionals are well positioned to support cessation efforts (30). Regular dental visits offer ideal opportunities to motivate patients and reinforce their confidence in quitting.

Ultimately, the convergence of behavioural theory, digital innovation and public policy holds promise for improving cessation outcomes. By embedding SE enhancement within multi-level interventions and spanning individual, interpersonal and environmental domains, vaping cessation efforts can transition from intention to sustained abstinence more effectively.

## Conclusion

This systematic review synthesises evidence from 11 studies to examine the relationship between SE and intention to quit vaping. Preliminary evidence suggests a predominantly positive association between these constructs, indicating that higher levels of confidence are generally linked to greater readiness to initiate cessation. However, the nature of this relationship is complex, as evidenced by the occasional negative associations potentially stemming from “optimistic bias” or overconfidence among certain young adult populations.

Significant measurement heterogeneity and current reliance on cross-sectional data, which prevent the inference of causality, are critical findings of this review. Furthermore, these findings should be interpreted as an initial foundation rather than a definitive proof of SE as a central predictor because only four studies explicitly tested the SE–ITQ association. Future research should prioritise longitudinal designs and the development of vaping-specific, validated instruments to better understand the temporal dynamics of SE. Clinically, these findings suggest that while enhancing a user’s confidence is a relevant component of cessation programmes, it must be integrated into broader behavioural strategies that address the unique delivery systems and patterns of e-cigarette use.

## Figures and Tables

**Figure 1:**
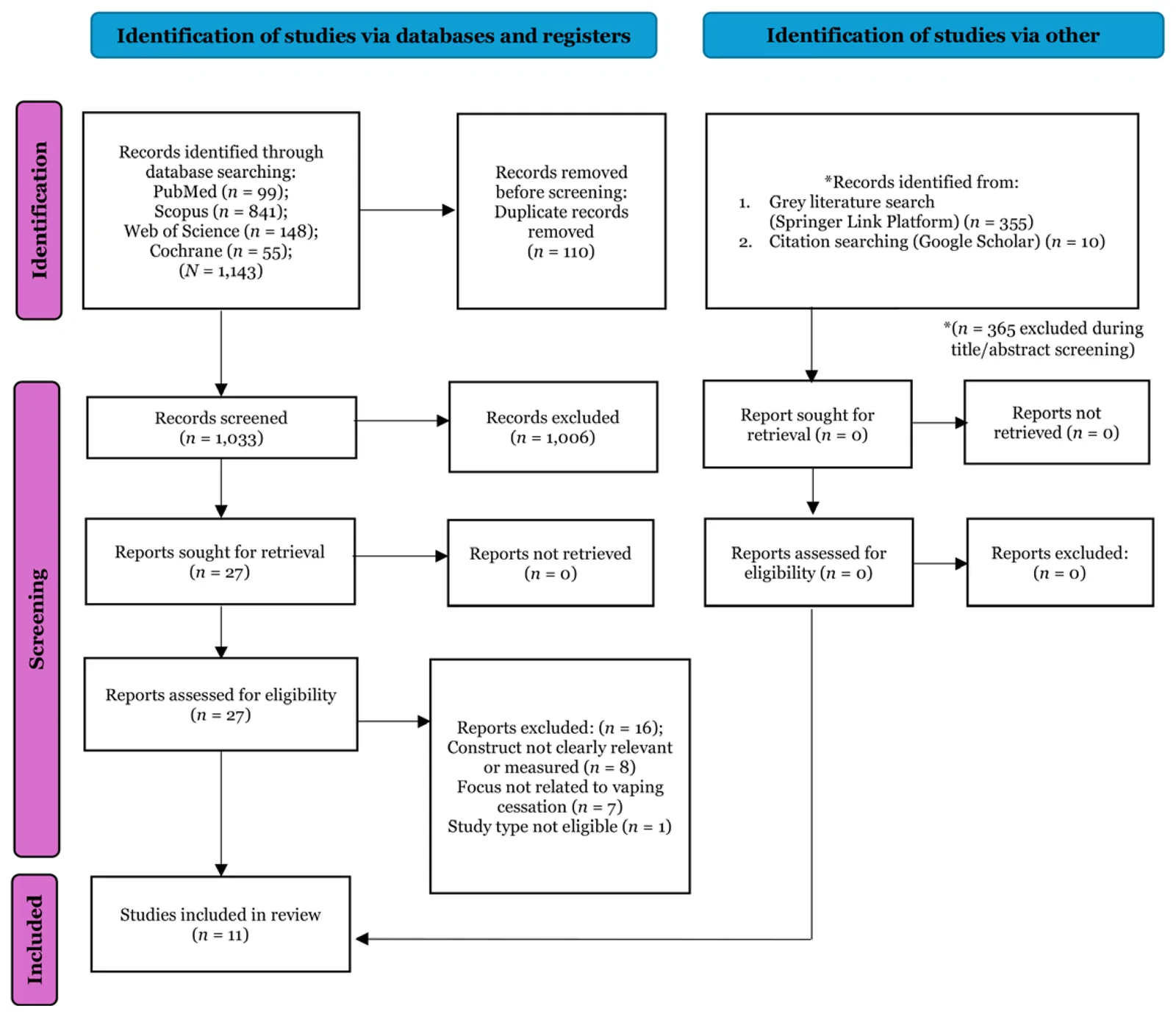
PRISMA 2020 flow diagram.

**Table 1:** Participant and methodological characteristics of included studies (*n* = 11).

No.	Author, Study design	Location, Participant numbers, Mean age	Gender distribution	Main aim and key findings related to ITQ and SE	Theoretical model
1.	Hobkirk et al. (5) Cross-sectional survey study utilising Amazon Mechanical Turk (MTurk) for data collection	United States *n* = 301 (81 never smoke, 55 current smokers, 165 former smoker) 31.9 (8.5 SD)	M% = 63.8 F% = 36.2 Others% = −	To examine perceptions of JUUL harm and addictiveness and identify factors associated with quit intentions, attempts, importance, and confidence among adult current JUUL users, who were categorised by their cigarette smoking status (never smoker, current smoker, and former smoker). Among participants, 22.6% expressed an ITQ JUUL within the next year, and the average SE (quit confidence) was 5.8 out of 10.	NR
2.	Romm et al. (4) Cross-sectional survey	United States *n* = 172 26.95 (4.91 SD)	M% = 41.9 F% = 57.6 Sexual minority = 47.7 (include gay/lesbian, bisexual, queer and another non-heterosexual identity)	To assess perceived challenges to quitting e-cigarettes, the impact of intervention messages on motivation and confidence (SE), and poly-use quitting preferences. Among participants, 39.0% were ready to quit e-cigarettes within six months, and quit confidence averaged 6.38 (3.14 SD) on a 10-point scale. Perceiving greater impact of messages targeting motivation was significantly associated with greater quitting importance (*P* < 0.001). Conversely, neither the perceived impact of messages targeting motivation nor confidence was associated with self-reported quit confidence (SE).	SCT
3.	Schoren et al. (22) Cross-sectional survey	Netherlands *n* = 477 (259 vapers, 135 smokers, 83 dual users) 37.1 (14.0 SD)	M% = 38.8 F% = 61.2 Others% = −	To assess vaping characteristics, reasons for use, and differences in motivational factors, including intentions to quit and SE, among smokers, vapers, and dual users using the I-Change Model. The study revealed that vapers had the highest SE scores on four of the 16 items, indicating they were significantly more confident in resisting vaping when seeing others vape, feeling sad, or feeling stressed/tensed (*P* < 0.001), and when craving nicotine (*P* < 0.05). Furthermore, the ITQ electronic cigarettes among dual users had a mean score of 4.4 (SD 2.9), while vapers had a mean score of 3.9 (SD 2.8). The difference was not statistically significant, measured on a scale from 1 (very weak) to 10 (very strong).	I-Change Model
4.	Le et al. (14) Qualitative semi-structured interviews	United States *n* = 40 26.30 (4.39 SD)	M% = 62.5 F% = 35.0 Others% = 2.5	NR	NR
	Le et al. (14) Quantitative survey	United States *n* = 726 24.15 (4.84 SD)	M% = 45.9 F% = 51.1 Others% = 3.0	To examine perceptions, motivators, and factors associated with e-cigarette cessation among young adults, utilising both quantitative measures (such as means for confidence in quitting and ITQ). The key outcomes of this study primarily focused on quantitative measures, including participants confidence and intentions related to e-cigarette cessation. The findings revealed that individuals reporting exclusive e-cigarette use had a higher mean confidence in quitting (7.59, SD = 3.06) compared to dual users (7.08, SD = 3.01), with this difference being statistically significant (*P* = 0.029). Regarding intentions to quit (measured as readiness to quit) within the next 30 days, about 17.8% of exclusive users and 17.5% of dual users expressed this intention, indicating similar cessation readiness across groups.	NR
5.	Romijnders et al. (15) Cross-sectional survey	Netherlands *n* = 116 (80 dual users, 36 exclusive e-cigarette users) 49.1 (12.5 SD)	M% = 43.1 F% = 56.9 Others% = −	The study aimed to identify a combination of smoking, product, and socio-cognitive factors that distinguish dual users from exclusive e-cigarette users using machine learning, to improve targeted health communication. Dual users have a higher mean intention to quit vaping within the next six months (3.8, SD = 2.0) compared to exclusive e-cigarette users (2.9, SD = 1.8). However, dual users report lower SE to not vape or smoke (3.9, SD = 1.2) than exclusive e-cigarette users (4.8, SD = 1.2).	NR
6.	Phua (23) Cross-sectional survey using online questionnaire	United States *n* = 1,016 41.6 (13.43 SD)	M% = 45.4 F% = 54.6 Others% = −	To examine the effect of participation in e-cigarette-related social media communities, subjective norms, and social identification on attitudes toward quitting, SE, and ITQ. Non-members of e-cigarette-related social media communities demonstrated significantly higher SE (5.58, SD = 0.81) and ITQ (4.12, SD = 0.89) compared to members (SE: 2.85, SD = 1.41; ITQ: 2.12, SD = 1.05). Participants exposed to negative subjective norms also showed higher SE (4.24, SD = 1.84) and ITQ (3.32, SD = 1.48) than those under positive norms. Similarly, individuals with low identification with other e-cigarette users reported greater SE (4.00, SD = 1.69) and ITQ (3.06, SD = 1.37) than those with high identification. The strongest ITQ was observed among participants with low social media use and negative subjective norms (4.37, SD = 0.77), while SE peaked among those with low social media use and high identification (5.67, SD = 0.77).	Ajzens TPB, Perkins and Berkowitzs Social Norms Approach, and Tajfel and Turners Social Identity Theory
7.	Wang-Schweig et al. (16) Cross-sectional survey	United States *n* = 163 (135 smokers and dual users, 28 vapers only) 39.3 (11.0 SD), smokers and dual users; 37.7 (7.4 SD), vapers only	M% = 46.7 (smokers and dual users); 78.6 (vapers only) F% = 53.3 (smokers and dual users); 21.4 (vapers only) Others% = −	To estimate the prevalence of tobacco, use among residents of Oxford House recovery homes and investigates predictors of future quitting intentions, including SE, motivation to quit, and nicotine dependence levels. It specifically compares two groups: residents who exclusively vape and those who only smoke or both smoke and vape (dual users). The mean SE to quit among vapers only was 2.05 (SE = 0.71), which was significantly lower than the mean of 3.60 (SE = 0.31) among cigarette smokers and dual users (*P* = 0.05). Regarding motivation to quit, vapers had a mean of 2.68 (SE = 0.79), also significantly lower than the 4.14 (SE = 0.35) reported by smokers and dual users (*P* = 0.05). These lower mean scores further support that vapers only have diminished confidence and motivation toward quitting tobacco; however, the significant difference in motivation disappeared once adjusted for sex.	NR
8.	Jun et al. (21) Cross-sectional, two-wave online survey (Wave 1 in 2020 and Wave 2 in 2021)	United States *n* = 165 (Wave 1) *n* = 347 (Wave 2) 21.55 (2.50 SD), Wave 1 21.74 (2.24 SD), Wave 2	M% = 50.9 (Wave 1); 43.5 (Wave 2) F% = 47.9 (Wave 1); 55.9 (Wave 2) Others% = 1.2 (Wave 1); 0.6 (Wave 2)	To investigate changes in young e-cigarette users perceptions of COVID-19 and traditional risks and benefits related to e-cigarette use and cessation during the pandemic, compare their evaluations of COVID-19 versus traditional threats, and examine how these perceptions influence their cessation intentions and attempts in conjunction with other factors. In Wave 1, 24.2% of young e-cigarette users planned to quit within six months, whereas in Wave 2, this increased to 30.8%. Regarding the Efficacy of Cessation (SE), the mean score was 3.51 (SD = 0.91) in Wave 1 and 3.59 (SD = 0.80) in Wave 2. The slight increase in confidence was not statistically significant( *P* = 0.315).	HBM
9.	Phua (17) Cross-sectional online survey	United States *n* = 1,016 41.6 (13.43 SD)	M% = 45.4 F% = 54.6 Others% = −	To examine how exposure to three types of Social Networking Sites-based e-cigarette marketing influences attitudes, behavioural control, ITQ, and SE, and how moderators (social identification, social comparison, . subjective norms) affect these relationships Exposure to SNS-based e-cigarette marketing significantly reduced both intentions to quit and SE (*P* < 0.001). Participants not exposed to marketing showed the highest ITQ (4.54, SD = 0.70) and SE (5.80, SD = 0.75), while those exposed to all marketing types had the lowest scores (intention: 1.33, SD = 0.49; SE: 1.77, SD = 0.51). Social identification further weakened these outcomes, indicating that higher exposure and stronger identification with e-cigarette users were linked to lower motivation and confidence to quit.	Ajzen’s TPB, Social Identity Theory (SIT)
10.	Rahman et al. (3) Cross-sectional survey	Australia *n* = 422 19.6 (1.7 SD)	M% = 18.5 F% = 67.8 Others% = 13.7	To examine, using the SCT framework, the association between SE, perceptions of e-cigarettes, and social norms with the intention to quit vaping. For the outcomes, the mean score for quit intention is 5.3 (2.4 SD) and the mean score for SE is 5.2 (2.7 SD). Specifically, in the hierarchical multiple regression analysis, SE was independently associated with quit intention with a standardised beta coefficient of 0.16( *P* ≤ 0.001), demonstrating that participants with greater confidence in their ability to quit are more likely to intend to cessation vaping.	SCT
11.	Sharma et al. (18) Cross-sectional study	United States *n* = 619 21.74 (1.6 SD)	M% = 46.7 F% = 52.0 Others% = 1.3	The study aimed to test the applicability of the MTM in predicting the initiation and sustenance of vaping cessation, hypothesising that all MTM subscales would independently predict quitting behaviour after controlling for demographic and behavioural factors. The study found that behavioural confidence was a significant predictor of intention to quit vaping, showing a strong positive association with other MTM constructs such as changes in the physical environment, emotional transformation, and practice for change (*P* < 0.001). In the hierarchical regression analysis, inclusion of behavioural confidence significantly improved the model’s prediction of quit initiation (Δ*R*^2^= 0.279), and the standardised beta coefficient in the final model indicated a strong positive association with initiation (*β* = 0.345, *P* < 0.001). This indicates that higher behavioural confidence was strongly linked to greater intention to initiate vaping cessation.	MTM

SE = self-efficacy; ITQ = intention to quit; MTM = Multi Theory Model; SCT = Social Cognitive Theory; TPB = Theory of Planned Behaviour; NR = not recorded.

**Table 2:** Details of the quality appraisal outcomes of each study.

Study	Inclusion criteria	Subjects and settings	Exposure measured	Condition criteria	Confounding identified	Confounding strategies	Outcomes measured	Statistical analysis	Overall risk of bias
Hobkirk et al. (5)	1	1	0	1	1	1	0	1	Low
Romm et al. (4)	1	1	1	1	1	1	0	1	Low
Schoren et al. (22)	1	1	1	1	1	1	0	1	Low
Le et al. (14)	1	1	* N/A	1	0	0	0	1	Moderate
Romijnders et al. (15)	1	1	1	1	1	1	0	1	Low
Phua (23)	1	1	1	1	0	0	1	1	Low
Wang-Schweig et al. (16)	1	1	0	1	1	1	0	1	Low
Jun et al. (21)	1	1	1	1	1	1	1	1	Low
Phua (17)	1	1	1	1	0	0	1	1	Low
Rahman et al. (3)	1	1	1	1	1	1	0	1	Low
Sharma et al. (18)	1	1	1	1	1	1	1	1	Low

* N/A = not applicable.

**Table 3:** Summary of SE instruments in the included studies.

No.	Study	Conceptualisation of SE	Scale type/ Response format	Reliability/ Statistical significance (Cronbach α or *P*-value)	Mean (SD/SE)	SE findings
1.	Hobkirk et al. (5)	Quit confidence as a perception variable, which can be interpreted as a measure of SE regarding quitting JUUL e-cigarette.	10-point Likert scale ranging from “not at all” (1) to “very” (10) Categorised into low (1–3), medium (4–7), and high (8–10) for analysis	*P*-value = 0.07	5.8 (SD 2.7)	Greater e-cigarette dependence, as assessed using the Penn State Electronic Cigarette Dependence Index, was linked to lower confidence in quitting. Overall, JUUL users demonstrated relatively low quit confidence, which was even lower among those with higher dependence levels.
2.	Romm et al. (4)	Perceived impact of intervention messages targeting confidence (related to SE to quit vaping).	7-point Likert scale: 1 = no impact at all; to 7 = a lot of impact	The Cronbach α for the aggregated impact of messages targeting confidence is 0.90	6.38 (SD 3.14)	The perceived impact of messages targeting confidence does not show a significant association with perceived quit confidence. Older age and more days of e-cigarette use were associated with lower self-reported quit confidence.
3.	Schoren et al. (22)	Confidence to resist vaping in various challenging situations (related to SE to refrain from vaping), such as when seeing others vape, feeling sad or stressed, experiencing cravings, waking up, being offered an e-cigarette, missing the flavour of cigarettes, or after eating.	5-point Likert scale for 16-items: 1 = definitely not; 5 = definitely	α = 0.95	Vapers showed higher confidence to resist vaping in situations such as: At a party = 3.8 (SD 1.3) While drinking alcohol = 3.5 (SD 1.4) When feeling frustrated or mad = 3.2 (SD 1.4) When seeing others vape = 3.6 (SD 1.3) When offered e-cigarettes socially = 3.5 (SD 1.2) They also felt confident to resist vaping when: Unsure how to act = 3.8 (SD 1.2) Feeling bored = 3.3 (SD 1.3) Feeling sad = 3.2 (SD 1.3) Feeling stressed = 3.0 (SD 1.3) Experiencing morning urges = 3.2 (SD 1.4) Craving nicotine = 2.8 (SD 1.3)	Vapers showed higher confidence in resisting vaping in various situations, such as when seeing others vape, feeling stressed or sad, craving nicotine, or receiving an e-cigarette offer, compared to smokers. Higher SE was linked to lower odds of smoking or dual use, indicating that individuals with greater confidence in quitting were less likely to smoke or use both products (cigarette and vape).
4.	Le et al. (14)	Perceived confidence in quitting e-cigarettes (related to SE to quit vaping), assessed through questions evaluating individuals’ belief in their ability to stop e-cigarette use, serving as measures of SE within the study context.	10-point Likert scale: 0 = not at all; to 10 = absolutely	*P* = 0.029	7.59 (SD 3.06) for exclusive e-cigarette users, 7.08 (SD 3.01) for dual users with other tobacco products	Confidence to quit was notably greater among exclusive e-cigarette users than among dual users. This indicates that dual users may have somewhat lower confidence in quitting, possibly due to the added difficulty of managing dependence on more than one nicotine product.
5.	Romijnders et al. (15)	Confidence in the ability to abstain from vaping and smoking, based on participants divided by two; dual users’ group and e-cigarette users.	7-point Likert scale: ranging from “completely disagree” (or “not confident at all”) to “completely agree” (or “completely confident”)	NR	3.9 (SD 1.2) for dual users’ group, 4.8 (SD 1.2) for e-cigarette users’ group	Exclusive e-cigarette users were more confident in their ability to refrain from vaping compared to dual users. SE to not vape and smoke is one of the seven most relevant factors distinguishing dual users from exclusive e-cigarette users, identified through random forest analysis with significant contribution to prediction accuracy (FDR 5%).
6.	Phua (23)	Individuals’ confidence to resist e-cigarette use in social settings.	19-item modified SE Scale; 7-point Likert scale ranging from “not confident at all” to “completely confident”	α = 0.96	5.67 (SD = 0.77) for participants with low social media use and high identification, and 5.47 (SD = 0.84) for those with low social media use and low identification	Higher social media engagement related to e-cigarettes, more positive social norms, and stronger social identification with users are all associated with reduced SE to quit e-cigarettes.
7.	Wang-Schweig et al. (16)	Participants’ current confidence in the ability to quit using vape.	Single-item Likert scale: ranging from “not confident at all” (0) to “extremely confident” (10)	*P* = 0.05	3.60 (SE 0.31) for smokers and dual users’ group, 2.05 (SE 0.71) for vapers only group	Individuals who exclusively vaped reported lower SE compared to smokers and dual users.
8.	Jun et al. (21)	Participants’ agreement with statements related to efficacy (confidence) to stop e-cigarette use.	6 statements on a 5-point Likert scale ranging from “strongly disagree” to “strongly agree”	α = 0.814 *P* = 0.315	3.51 (SD 0.91) for Wave 1; 3.59 (SD 0.80) for Wave 2	Greater SE was negatively associated with intentions to quit; however. This suggests that young e-cigarette users’ high confidence in their ability to quit may hinder them from planning to quit. Providing realistic assessments of SE could therefore improve cessation support.
9.	Phua (17)	Participants’ confidence in resisting e-cigarette use in challenging situations (e.g. feeling nervous or socialising) and examined how exposure to e-cigarette marketing on social networking sites affected their confidence to quit.	19-item of 7-point Likert scale ranging from “not confident at all” to “completely confident”	α = 0.96	Not Exposed: 5.80 (SD = 0.75) Saw ads only: Mean = 5.27 (SD = 0.79) Followed brand pages only: 4.38 (SD = 0.80) Saw ads + Followed brand pages: 4.18 (SD = 0.98) Joined user-created pages: 2.71 (SD = 1.49) Saw ads + Joined user groups: 2.22 (SD = 0.67) Followed brand pages + joined user groups: 1.89 (SD = 0.60) Exposed to all three types of marketing: 1.77 (SD = 0.51)	Current e-cigarette users who participate in user-created social networking site communities showed significantly lower cessation self-efficacy. Those exposed to all three types of e-cigarette marketing messages on SNS within the past month demonstrated the lowest confidence in their ability to quit.
10.	Rahman et al. (3)	Participants’ confidence in quitting e-cigarettes, assessed by rating how confident they were in their ability to quit vaping at the present moment.	Single-item 10-point Likert scale ranging from “not at all confident” (1) to “extremely confident” (10)	NR	5.2 (SD 2.7)	SE was found to be a significant factor influencing the intention to quit vaping and should be considered a key element in designing effective vaping cessation interventions.
11.	Sharma et al.(18)	Participants’ confidence in quitting e-cigarettes, assessed using the MTM behavioural confidence subscale – a tool that has initiation and sustenance components.	5-point Likert scale called the surety scale (“not at all sure” to “completely sure”)	α = 0.91	8.84 (SD 6.02)	Higher behavioural confidence was significantly associated with stronger initiation of quitting behaviour.

SE = self-efficacy; MTM = Multi Theory Model; NR = not recorded.

**Table 4:** Summary of ITQ instruments in the included studies.

No.	Study	Conceptualisation of ITQ	Scale type/ Response format	Reliability/ Statistical significance (Cronbach α or *P*-value)	%/ Mean (SD)	ITQ findings	ITQ–SE analysis
1.	Hobkirk et al. (5)	A single-item question asking participants whether they planned to continue using JUUL for at least the next year or quit within that time frame.	Single-item 3-point categorical scale assessing JUUL use intentions, with response options: “I’ll quit using JUUL within a year,” “I plan to continue using JUUL,” and “Don’t know.”	NR	22.6% will quit within a year, 77.4% will continue to use/ don’t know	The primary significant factor was the perception of the e-cigarette’s addictiveness. Compared to users who found JUUL “Not at all addictive” (the reference group), those who found it “Moderately addictive” were 3.36 times more likely to intend to quit (OR = 3.36). This likelihood dramatically increased for those who found it “Very addictive,” making them 12.46 times more likely to intend to quit (OR = 12.46). Additionally, the frequency of use was also a significant factor, with fewer JUUL use times per day being associated with a greater ITQ (OR = 0.70). Notably, the Penn State Electronic Cigarette Dependence Index score, a measure of dependence, was not a significant correlate in the final model for quit intentions.	No
2.	Romm et al. (4)	The measure of readiness to quit serves as an indicator of ITQ. Participants reported their preparedness to cease e-cigarette use within specific timeframes: within the next 30 days, within the next six months, in more than six months, or not thinking about quitting.	4-point categorical scale with response options: “Within the next 30 days,” “Within the next six months,” “In more than six months,” and “Not thinking about quitting.”	NR	16.3% shows readiness to quit in next 30 days, while 39.0% in next six months	55.3% of participants reported readiness to quit overall.	No
3.	Schoren et al. (22)	The ITQ electronic and/or conventional cigarettes was assessed using two items based on how strongly they intended to stop smoking conventional cigarettes and e-cigarettes.	10-point Likert scale ranging from “very weak” (1) to “very strong” (10) for ITQ conventional cigarettes and e-cigarettes.	*P*-value > 0.05	4.4 (SD 2.9) for dual users, and 3.9 (SD 2.8) for vapers only	Mean ITQ e-cigarettes was somewhat higher in dual users than in vapers only, although the difference was not statistically significant.	No
4.	Le et al. (14)	The measure of readiness to quit serves as an indicator of ITQ. Participants reported their preparedness to cease e-cigarette use within specific timeframes: within the next 30 days, within the next six months, in more than six months, or not thinking about quitting.	4-point categorical scale with response options: “Yes, within the next 30 days,” “Yes, within the next 6 months,” “Yes, in more than 6 months,” and “Not thinking about quitting.”	*P* = 0.906	Exclusive e-cigarette users: 17.8% next 30 days, 19.0% next six months, 18.6% more than six months, 44.6% not thinking of quitting Dual users: 17.5% next 30 days, 21.2% next six months, 17.3% more than six months, 44.0% not thinking about quitting	The majority of both exclusive e-cigarette users and dual users were not thinking about quitting, while smaller proportions reported readiness to quit within 30 days, six months, or more than six months. Overall, readiness to quit was relatively low and similar across both groups, with no significant difference between them.	No
5.	Romijnders et al. (15)	Intention to quit vaping was measured in two forms: ITQ (A) participants rated their likelihood of quitting within the next six months on a 7-point scale, and ITQ (B) participants selected the statement that best reflected their attitude toward stopping vaping.	7-point Likert scale with responses from “totally disagree” (1) to “totally agree” (7) or semantic-differentials.		ITQ (A): 3.8 (SD 2.0) for dual users, 2.9 (SD 1.8) for e-cigarette users ITQ (B): 3.6 (SD 2.4) for dual users, 2.3 (SD 1.8) for e-cigarette users	Dual users exhibited a higher mean intention to quit vaping. The ITQ scores were among the significant factors in distinguishing dual users from exclusive e-cigarette users in the machine learning model.	No
6.	Phua (23)	ITQ e-cigarette was measured under factors such as social media community (SMC) membership and active participation, subjective norms related to e-cigarettes, and social identification with other e-cigarette users.	7-point Likert scales, including statements such as “During the next 6 months, I intend to quit using e-cigarettes” and “During the next 6 months, I will quit using e-cigarettes.”	α = 0.98	SMCs membership: 4.12 (SD 0.89) for not members; 2.12 (SD 1.05) for members Effect of subjective norms: 3.32 (SD 1.48) for – ve subjective norm; 2.48 (SD 1.23) for +ve subjective norm Effect of social identification: 3.06 (SD 1.37) for low identification: 2.55 (SD 1.33) for high identification	People who were not members of e-cigarette SMCs reported a higher ITQ than those who were members. Similarly, participants experiencing negative subjective norms had higher ITQ compared with those experiencing positive norms, and those with low social identification with other e-cigarette users reported higher ITQ than those with high social identification.	No
7.	Wang-Schweig et al. (16)	ITQ was assessed based on whether participants were seriously considering stopping tobacco use, including vaping, within the next six months.	Binary yes/no format.	*P* < 0.05	37.3% for smokers and dual users, 15.4% for vapers only	Vapers showed lower ITQ tobacco use within the next six months compared with smokers and dual users.	Yes
8.	Jun et al. (21)	Assessed through cessation intent, where participants indicated whether they had no plan, were unsure, or planned to quit e-cigarette use within the next month/ six months.	Using a categorical measure with five response options: “I have no plan to quit vaping,” “I plan to quit vaping in the next month,” . next six months: significant “I plan to quit vaping in the next 6 months,” “I plan to quit vaping in the future, after 6 months,” and “I don’t know.”	*P* < 0.092	(Wave 1 vs. Wave 2) No plan to quit vaping: 24.8% → 21.6% Plan to quit in the next month: 20.0% → 25.1% Plan to quit in the 24.2% → 30.8% Plan to quit after six months: 17.6% → 15.0% Don’t know: 13.3% → 7.5%	Cessation intent showed a slight increase from Wave 1 to Wave 2, with more participants planning to quit within the next month or six months and fewer reporting uncertainty or no plans to quit. However, the overall difference between waves was not statistically	Yes
9.	Phua (17)	ITQ was conceptualised as participants’ determination to stop using e-cigarettes such as “During the next six months, I intend to quit smoking e-cigarettes,” across varying levels of social networking site–based e-cigarette marketing exposure.	7-point Likert scales, including statements such as “During the next 6 months, I intend to quit using e-cigarettes” and “During the next 6 months, I will quit using e-cigarettes.”	α = 0.98	Not exposed: 4.54 (SD 0.70) Saw ads: 3.54 (SD 0.79) Followed brand pages: 3.34 (SD 0.84) Saw ads + brand pages: 3.01 (SD 0.69) Joined user-created pages: 2.57 (SD 1.27) Saw ads + user-created groups: 2.04 (SD 0.71) Brand pages + user-created groups: 1.44 (SD 0.53) Exposed to all three types: 1.33 (SD 0.49)	ITQ significantly decreased with increasing exposure to SNS-based e-cigarette marketing. Those not exposed reported the highest quitting intention, while those exposed to all three types of content (ads, brand pages, and user-created groups) reported the lowest.	No
10.	Rahman et al. (3)	Mentioned as quit intention, which was measured through a validated 11-point Contemplation Ladder.	10-point scale, ranging from “no thought of quitting” (1) to “taking action to quit.” The scale was treated as a continuous variable for analysis.	NR	5.3 (SD 2.4)	Hierarchical regression analysis indicated that, aside from baseline quit intention, self-efficacy had a significant positive association with quit intention, emphasising its crucial role in vaping cessation interventions.	Yes
11.	Sharma et al. (18)	ITQ was treated as the dependent variable in the analysis, functioning as an outcome variable for both the initiation and sustenance of vaping cessation behaviour, based on the MTM.	5-point likelihood scale, ranging from “not at all likely” to “completely likely.”	Predictive effect of all MTM constructs was *P* < 0.001 (for both initiation and sustenance)	Initiation: 1.89 (SD 1.32); Sustenance: 1.74 (SD 1.35)	Behavioural confidence was the strongest predictor for initiation. Emotional transformation was the strongest predictor for sustenance. All MTM constructs were significant predictors.	Yes

SE = self-efficacy; MTM = Multi Theory Model; ITQ = intention to quit; NR = not recorded.

**Table 5:** Relationship between SE and ITQ

No.	Study	Theoretical model	Statistical test	Main findings (direction and nature of SE–ITQ relationship)	Significance (P/*β*/r)
1.	Wang-Schweig et al. (16)	NR	Multiple logistic regression analysis was used to examine the relationship between SE and the ITQ e-cigarette use within the past six months, adjusting for sociodemographic variables.	Direction: Positive association; higher SE increased the likelihood of intending to quit. Strength: The adjusted odds ratio (OR) was 1.47, indicating that for each unit increase in SE, the odds of intending to quit increased by approximately 47%.	The relationship was statistically significant with *P* = 0.03, and *β* (regression coefficient) = 0.385.
2.	Jun et al. (21)	HBM	The hierarchical regression analysis examining the influence of self-efficacy on cessation intention controlled for other variables like sociodemographic variables, HBM variables, e-cigarette dependence, poly-tobacco use, and COVID-19-related perceptions.	Wave 1, Model 1 Direction: Negative association. Higher SE was linked with a minimally weaker ITQ. Strength: The relationship indicated only a minimal, non-significant effect on quit intention. Wave 1, Model 2 Direction: Negative association; higher SE corresponded to a slightly reduced cessation intent. Strength: The relationship showed a weak, non-significant negative effect on cessation intent. Wave 2, Model 1 Direction: Negative association; higher SE significantly predicted a lower likelihood of intending to quit. Strength: The relationship showed a significant negative effect on ITQ. Wave 2, Model 2 Direction: Negative association; higher SE was significantly associated with decreased ITQ. Strength: The relationship indicated a significant inverse effect.	Wave 1, Model 1 *β* = −0.040; not statistically significant Wave 1, Model 2 *β* = −0.056; not statistically significant Wave 2, Model 1 *β* = −0.139; statistically significant at *P* < 0.05. Wave 2, Model 2 *β* = −0.153; statistically significant at *P* < 0.05
3.	Rahman et al. (3)	SCT	Hierarchical regression is used to evaluate the additional explanatory power of SCT constructs (including SE) on quit intention.	Direction: Positive association; higher SE was associated with increased ITQ. Strength: The relationship was statistically significant with a small effect size.	*β* = 0.16; statistically significant at *P* < 0.001
4.	Sharma et al. (18)	MTM	Hierarchical multiple regression analysis was performed to evaluate the predictive power of behavioural confidence on the intention of the initiation of quitting behaviour model under MTM.	Direction: Positive association; higher behavioural confidence was associated with increased likelihood of vaping quitting behaviour. Strength: Behavioural confidence was the strongest predictor in the initiation model.	In Model 3, the effect was strong (*β* = 0.560). In the final model (Model 4), the unique standardised coefficient was *β* = 0.345, indicating a significant positive association between higher behavioural confidence and greater intention to initiate vaping quitting behaviour. Behavioural confidence remained highly significant across models (*P* < 0.001), confirming its strong predictive value for initiating vaping quitting behaviour.

SE = self-efficacy; ITQ = intention to quit; HBM = Health Belief Model; MTM = Multi Theory Model; SCT = Social Cognitive Theory; NR = not recorded.

## Appendices

**Appendix 1:** Search strategy.

Part A. Primary bibliographic databases (systematic search). These searches were conducted from inception until 1 November 2025.	
Database	Search string
Cochrane	“self-efficacy” OR “self efficacy” OR “quit confidence” OR confidence OR “perceived control” AND “intention to quit” OR “quit intention” OR “readiness to quit” OR “motivation to quit” AND vape* OR vaping OR vaper* OR (e NEXT cigarette*) OR (electronic NEXT cigarette*) OR (electronic NEXT nicotine NEXT delivery NEXT system*) OR ENDS OR JUUL OR (vape NEXT pod) OR (vape NEXT pen)
Web of Science (WoS)	(“self-efficacy” OR “self efficacy” OR “quit confidence” OR confidence OR “perceived control”) AND (“intention to quit” OR “quit intention” OR “readiness to quit” OR “motivation to quit”) AND (vaping OR vape OR vapes OR “e-cigarette” OR “electronic cigarette” OR “electronic nicotine delivery system” OR ENDS OR JUUL OR “vape pod” OR “vape pen”)
PubMed	(“Electronic Nicotine Delivery Systems” [MeSH] OR Vaping OR “e-cigarette*” OR “electronic cigarette*”) AND (“Self Efficacy” [MeSH] OR “Self-Efficacy” OR confidence OR “perceived control” OR “quit confidence”) AND (“Intention” [MeSH] OR “Intention to Quit” OR “Quit Intention” OR “Readiness to Quit” OR “Motivation to Quit” OR “Quit Attempts”)
Scopus	(“self-efficacy” OR “self efficacy” OR confidence OR “perceived control”) AND (“intention to quit” OR “quit intention” OR “quit attempts”) AND (vaping OR “e-cigarette” OR “electronic cigarette” OR JUUL OR “vape pod” OR “vape pen” OR ecig OR ENDS)

Part B: Manual and citation searching (other methods).		
Source	Search date	Search method/strategy
Springer Link	From inception until 1 November 2025	Manual Grey Literature Search: Used tailored Boolean strings: (“self-efficacy” OR “self efficacy” OR “quit confidence” OR confidence OR “perceived control”) AND (“intention to quit” OR “quit intention” OR “readiness to quit” OR “motivation to quit”) AND (vaping OR vape OR “e-cigarette” OR “electronic cigarette” OR “electronic nicotine delivery system” OR ENDS OR JUUL OR “vape pod” OR “vape pen”)
Google Scholar	Last search on 12 November 2025	Citation Searching: Manual backward citation tracking (reference list screening) and forward citation tracking (cited-by analysis) of all studies identified for inclusion.
